# Post-Validation Survey in Two Districts of Morocco after the Elimination of Trachoma as a Public Health Problem

**DOI:** 10.4269/ajtmh.21-1140

**Published:** 2022-03-28

**Authors:** Jaouad Hammou, Sarah Anne J. Guagliardo, Majdouline Obtel, Rachid Razine, Abbas Ermilo Haroun, Mohamed Youbi, Abdelkrim Meziane Bellefquih, Michael White, Sarah Gwyn, Diana L. Martin

**Affiliations:** ^1^ Faculty of Medicine and Pharmacy of Rabat, Mohammed V University, Rabat, Morocco;; ^2^ Division of Parasitic Diseases and Malaria, Centers for Disease Control and Prevention, Atlanta, Georgia;; ^3^ Laboratory of Biostatistics, Clinical Research and Epidemiology, Department of Public Health, Faculty of Medicine and Pharmacy of Rabat, Mohammed V University, Rabat, Morocco;; ^4^ Laboratory of Community Heath (Public Health, Preventive Medicine and Hygiene), Department of Public Health, Faculty of Medicine and Pharmacy, University Mohammed V, Rabat, Morocco;; ^5^ Directorate of Epidemiology and Disease Control, Ministry of Health, Rabat, Morocco;; ^6^ Infectious Disease Epidemiology and Analytics Unit, Department of Global Health, Institute Pasteur, Paris, France

## Abstract

Trachoma is the leading infectious cause of blindness. In 2016, Morocco was validated by WHO as having eliminated trachoma as a public health problem. We evaluated two previously endemic districts in Morocco for trachomatous inflammation—follicular (TF), trachomatous trichiasis (TT), and antibodies against *Chlamydia trachomatis*, the causative agent of trachoma. Community-based cross-sectional surveys in the districts of Boumalene Dades and Agdez included 4,445 participants for whom both questionnaire and serology data were available; 58% were aged 1–9 years. Participants had eyes examined for TF and blood collected for analysis of antibodies to the *C. trachomatis* antigen Pgp3 by both a multiplex bead assay (MBA) and lateral flow assay (LFA). Seroconversion rates (SCR) per 100 people per year were used to estimate changes in the force of infection using Bayesian serocatalytic models. In Agdez, TF prevalence in 1–9-year-olds was 0.3%, seroprevalence ranged from 9.4% to 11.4%, and SCR estimates ranged from 2.4 to 3.0. In Boumalene Dades, TF prevalence in 1–9-year-olds was 0.07%, and modeling data from the different assays indicated a decrease in transmission between 20 and 24 years ago. The TF data support an absence of active trachoma in the two districts examined. However, seroprevalence and SCR in younger people were higher in Agdez than Boumalene Dades, showing that there can be differences in serology metrics in areas with similar TF prevalence. Data will be included in multicountry analyses to better understand potential thresholds for serological surveillance in trachoma.

## INTRODUCTION

Trachoma, caused by ocular serovars of *Chlamydia trachomatis*, is the leading infectious cause of blindness,^1^^,^^2^ producing blindness in an estimated 1.3 million people worldwide. Africa is the most heavily affected continent. Despite this heavy burden, the past 15–20 years have seen major progress toward elimination of trachoma as a public health problem. The number of people at risk of trachoma worldwide has fallen from 1.5 billion in 2002 to 137 million in 2020, a reduction of 91%.^3^ Since 2011, 11 countries, including Morocco, have been validated by WHO as having eliminated trachoma as a public health problem.^4^

The WHO simplified grading system for trachoma uses five signs to facilitate standardized reporting: trachomatous inflammation—follicular (TF), trachomatous inflammation—intense (TI), trachomatous scarring (TS), trachomatous trichiasis (TT), and corneal opacity (CO).^5^ Trachoma first manifests as inflammation of the conjunctiva. The signs of active trachoma, TF and TI, occur primarily in young children. Recurrence and persistence of active trachoma can lead to TS and the development of TT, in which the eyelashes turn inward, causing them to rub against the eyeball. Trachomatous trichiasis is extremely painful,^6^ but can be corrected surgically. If left untreated, it can lead to corneal opacification, low vision, and blindness. Modeling suggests that at least 150 lifetime conjunctival *C. trachomatis* infections are necessary to develop TT.^7^

The main known risk factors for trachoma are poverty, close contact with others who have active trachoma, overcrowded conditions, dirty faces, inadequate access to water and sanitation, presence of flies, and migration of infected persons exposing host communities to new bacterial strains.^8^^,^^9^ Programs seeking to eliminate trachoma therefore address these risk factors through a set of interventions known as the “SAFE strategy,” comprising (S) surgery for TT, (A) antibiotics to clear ocular *C. trachomatis* infection, (F) facial cleanliness, and (E) environmental improvement, particularly improved access to water and sanitation.^10^ Through this multifaceted approach to cure and prevent disease, the S component of the SAFE strategy is aimed at individuals, whereas A, F, and E are community-based interventions targeting entire populations.

As early as the 1950s, Morocco’s Ministry of Health and National Ophthalmology Center provided field-based care for trachoma in disadvantaged communities. Upon endorsement of the SAFE strategy by WHO in the 1990s,^11^ Morocco began to fully implement it in the provinces that were still affected, including Errachidia, Figuig, Tata, Zagora, and Ouarzazate. By 2005, all previously endemic areas had reached the TF elimination prevalence threshold of < 5% in 1–9-year-olds, as defined by WHO. In 2008, all 37 districts (split from 18 parent districts) in Morocco were declared to have achieved this TF threshold, and mass distribution of antibiotics was halted. Surveillance activities began in 2007 and a validation survey was conducted in 2009. In November 2016, Morocco was recognized by WHO as having eliminated trachoma as a public health problem.^4^

There are currently no WHO recommendations for post-elimination surveillance for trachoma to ensure the disease does not recrudesce once the full SAFE strategy is no longer in place. The use of serological tests provides an opportunity to determine population-level exposure to infection, and when combined with age, can potentially give a useful indicator of transmission intensity over time. The prevalence of antibodies to the *C. trachomatis* antigens Pgp3 and CT694^12^^–^^14^ increase with age in children living in trachoma-endemic communities,^14^^,^^15^ but not in areas of low or no transmission.^16^^–^^18^ Antibodies could therefore be used to differentiate high-prevalence settings for trachoma, in which children are repeatedly infected with ocular *C. trachomatis* throughout childhood, compared with low-prevalence settings for trachoma in which children might only be infected once or a few times during childhood. These data are consistent when using different testing platforms.^16^^,^^19^ Further research is required to refine interpretation and guide the potential application of data on antibodies to *C. trachomatis* in programmatic decision-making.

We conducted a survey in September 2019 to determine the prevalence of trachoma and to compare serological prevalence to TF prevalence in two previously endemic districts Morocco: Boumalene Dades, Tinghir Province^20^ and Agdez, Zagora Province. These districts were selected based on the historical prevalence in the respected districts. The previous province where Boumalene Dades was located, Ouarzazate (Tinghir split from Ouarzazate in 2009) reached TF < 5% by 1997.^20^ Agdez had a TF prevalence of 8.8% in a 2004 survey and did not reach TF < 5% until 2005.^20^ Results will provide the Moroccan Ministry of Health with evidence as to whether these districts have sustained their elimination targets for trachoma in the selected regions. Results will also further inform the WHO Alliance for the Global Elimination of Trachoma by 2020 (GET2020) on the potentially utility of sero-surveillance in the post-validation setting.

## MATERIALS AND METHODS

### Ethics.

The risks and benefits of participating in the study were explained to participants prior to enrollment. Written informed consent of the household head and each study participant was obtained before data and specimen collection began. Parental consent was obtained from all minors and assent from older children was obtained. The informed consent form and information sheets were translated into the local language. The protocol was approved by the Biomedical Research Ethics Committee at the University Mohammed Soussi—V in Morocco. Staff at the U.S. Centers for Disease Control and Prevention did not interact with participants or have access to identifying information.

### Study sites.

**Figure 1.**
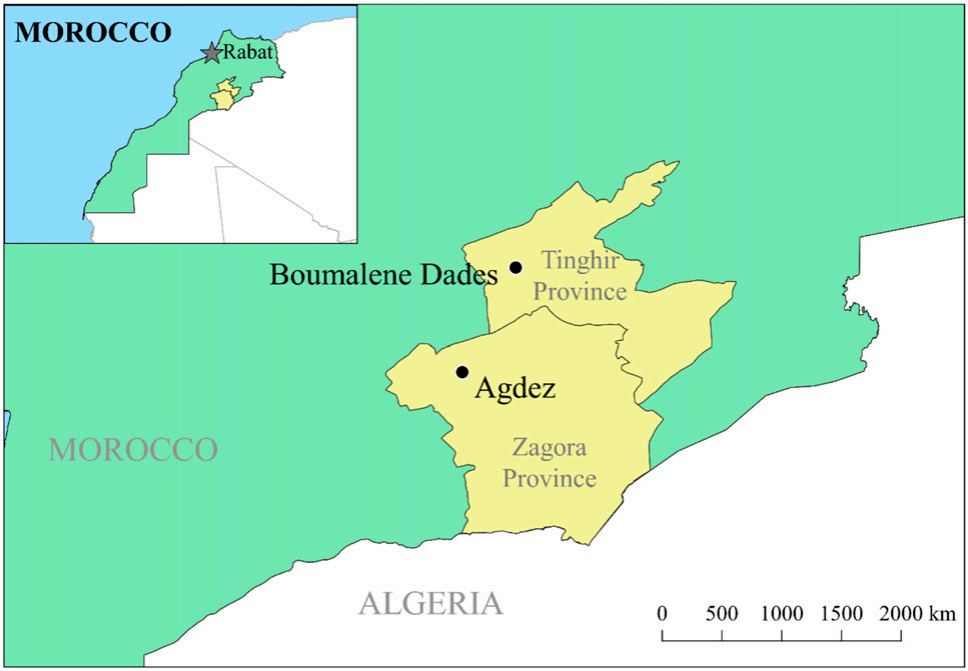
Map of study locations. The study sites included Agdez District (Zagora Province) and Boumalene Dades (Tinghir Province). Morocco.

The study was conducted in the provinces of Tinghir and Zagora, where trachoma was endemic in the 1990s. In the early 1990s, these provinces were part of the province of Ouarzazate. In 1997, the province of Zagora was formed and had a TF prevalence of approximately 60% in 1997, whereas Ouarzazate achieved the elimination threshold of < 5% TF by 1997. Zagora did not achieve a TF prevalence < 5% until 2005.^20^ Tinghir was split from Ouarzarzate in 2009. For this survey, one district from each of these provinces was selected arbitrarily: Agdez district in Zagora province and Boumalene Dades district in Tinghir province (Figure 1).

### Study design and sample size calculations.

This study used a two-stage cluster sampling design. We selected 30 clusters (i.e., villages) per district using probability proportional to size sampling. Data from the Statistics Directorate, Office of the High Commissioner for Planning were used to estimate the number of children in the household in each of the provinces. In the second stage, households per cluster were then selected from an exhaustive list of survey districts selected for the General Census of Population and Housing carried out in September 2004 using compact segment sampling, with a target of 20 households per cluster in Boumalene Dades and 33 households per cluster in Agdez. Within selected households, all individuals aged 1 year or above were eligible to participate. Sample size calculations assumed a TF prevalence in 1–9-year-olds of 4% ± 1% TF, an α error of 95% and a design effect for cluster sampling of 2. We calculated target sample sizes of 1,200 children aged 1–9 years and 2,020 persons aged 15 and older for each district.

### Data collection.

Each participant was asked to complete a questionnaire, to have their eyes examined, and to have their finger pricked for blood collection for antibody testing. Interviews were conducted by experienced qualitative researchers. Interview questions collected information about household-level demographic information as well as household access to water and a latrine. The head of the household generally responded to the questionnaire; when the head of household was not available, another adult was asked to complete it. Questionnaires and clinical examination results were collected on paper forms and later entered into a Microsoft Excel workbook in preparation for analysis.

All consenting individuals aged ≥ 1 year in every selected household had their eyes examined for clinical signs of trachoma (TF, TI, TS, TT, CO) based on the WHO Simplified Grading Scale by licensed ophthalmologists.^21^ Each district had a single team consisting of one ophthalmologist and one nurse conducting clinical testing. Facial cleanliness was assessed by looking for traces of eye discharge or nasal discharge on the upper lips or cheeks.

### Specimen collection and processing.

Finger prick blood was collected onto filter paper discs with six circular extensions, each calibrated to absorb 10 µL of whole blood (TropBio Pty Ltd., Townsville, Queensland, Australia). Each filter paper was labeled with a unique barcode, air dried for at least 2 hours, and then placed into individual sealable plastic bags. Dried blood spots (DBS) were transported at room temperature within 7 days of collection and stored at −20°C until testing. Trainings were held on DBS collection and storage in advance of field work to ensure correct and uniform sample collection. Dried blood spots were tested for antibodies against the *C. trachomatis* antigens Pgp3 and CT694 using the multiplex bead assay (MBA) at the U.S. Centers for Disease Control and Prevention (CDC), Atlanta.^12^ Dried blood spots were also tested for antibodies to Pgp3 using a lateral-flow assay (LFA).^16^

### Determination of cut-off values.

Cutoffs for seropositivity were determined using receiver operating characteristic curve analysis on a positive panel of 101 samples from ocular *C. trachomatis* PCR-positive individuals from the United Republic of Tanzania and negative panel of 74 pediatric samples from New York, NY. The cutoff for positivity was established as an MFI-bg (median fluorescence intensity minus background) value of 1,624 for Pgp3 and 347 for CT694.

### Data analysis.

Data were imported into R 2.2.1^22^ for analysis and cleaning. We used data from questionnaires (*N* = 5,431) to test for differences between the two study populations in terms of individual characteristics (sex, facial cleanliness among 1–9-year-olds) and household characteristics (access to a latrine, access to potable water) characteristics (Chi-square/Fisher’s exact tests, significance cut off *P* < 0.05). Median age between the two districts was evaluated using Wilcoxon rank-sum test.

Using census data from Morocco, we calculated the age-adjusted district-level prevalence of TF, TS, TT, and CO by age group (1–9 years, ≥ 15 years, and all ages).^23^^,^^24^ No instances of TI were observed; therefore, no analysis was conducted for this sign. The 95% CIs of the cluster means were calculated via bootstrapping over 10,000 iterations. To compare the overlap in trachoma grading indicators, we developed a Venn diagram of people presenting with TF, TS, TT, and CO. We also compared trachoma grading indicators with serostatus by calculating the proportion of people with each of the trachoma indicators who were seropositive by Pgp3 MBA, CT694 MBA, and Pgp3 LFA.

Serology data from MBA and LFA were merged to include only participants who had results for both tests available as well as demographic data (*N* = 4,445). There were no significant demographic differences between participants with both an MBA and LFA result and participants who had results from only one assay. To characterize differences in intensity of antibody levels by age among children 1–9 years and among all participants by study site, we graphed MFI data from MBA and used Wilcoxon tests to compare median MFI in Agdez versus Boumalene Dades (*P* < 0.05). Age-adjusted seroprevalence generated from Pgp3 MBA and CT694 MBA and Pgp3 LFA was calculated by age group in 5-year intervals, and the 95% CIs (using Wilson’s score interval) were obtained.

We estimated seroconversion rates for both assays in both districts using Bayesian serocatalytic models, which characterize the change in proportion of seropositive individuals by age.^25^^–^^27^ Following the methods described in Pinsent et al, we considered two transmission scenarios including 1) a constant rate of transmission (Model 1), and 2) a drop in transmission at a fixed time point (Model 2).^25^ We used Markov Chain Monte Carlo to estimate the median and 95% credible intervals (CrI) for model parameters. An informative prior was used to estimate the seroreversion rate, rho (Pgp3 ∼ N(0.26, 0.003); CT694 ∼ N(0.017,0.002)), based on previously published works.^25^ A list of parameters contained in each of the two modeling scenarios is shown in Supplemental Table 1. The Gelman-Rubin statistic < 1.1^28^ and effective sample size (ESS) > 300 were used to evaluate chain convergence. For children aged 1–9 years, we conducted Model 1, constant rate of transmission, because community mass drug administration (MDA) programs for trachoma were halted in 2008 in Morocco, before these children were born. For participants of all ages, we ran both the constant transmission model and the decline in transmission model to account for the impact of MDA programs in Morocco. The two models for all ages were compared by the Deviance Information Criterion to determine the most likely scenario given the observed data. Seroconversion rates (SCR) and seroreversion rates (SRR) were scaled to 100 people per year for ease of interpretation (e.g., an SCR of 0.02 was presented as 2).

## RESULTS

### Study population.

During September 2019, a total of 5,341 persons sampled were sampled. The average age of participants was 20.6 years (median: 9 years), and the age distribution varied significantly by district, with Adgez having older participants (Adgez median: 13 years, Boumalene Dades median: 7 years [*P* < 0.001]). Approximately 57% of participants were female, and participation by gender did not vary significantly between the two districts (Table 1). Among 2,617 children aged 1–9 years, 99.9% had clean faces; all nine children without clean faces were in Agdez. Multiplex bead assay testing was conducted among approximately 84% of participants (*N* = 4445), with higher rates of participation among those from Adgez (*P* < 0.0001).

**Table 1:** Characteristics of study participants in Agdez and Boumalene Dades districts, Morocco

	Overall		Agdez		Boumalene Dades			
	*N* = 5,431		*N* = 2,940 (54.1%)		*N* = 2,491 (45.8%)			
	N	(%)	n	(%)	n	(%)	χ^2^	*P*
**Sex**								
Female	3.093	(56.9)	1,702	(57.9)	1,391	(55.8)		
Male	2,338	(43.0)	1,238	(42.1)	1,100	(44.2)		
							2.2	0.14
**Age**								
1–9	2,617	(48.4)	1,264	(43.0)	1,353	(54.3)		
> 9	2,796	(51.6)	1,668	(56.7)	1,128	(45.3)		
Missing	18						69.1	**< 0.0001**
**Serologic test result available (MBA and LFA)**								
Yes	4,445	(81.8)	2,565	(87.2)	1,880	(75.5)		
No*	986	(18.2)	375	(12.8)	611	(24.5)		
							125.8	**< 0.0001**

LFA = lateral flow assay; MBA = multiplex bead assay. Bolded *p*-values indicate statistical significance. Among children aged 1–9 (*N* = 2,617).

* Serologic testing results are only presented when demographic data (e.g., age) were also available.

The overall median household size was four, and household size varied significantly by district (Agdez median: five persons, Boumalene Dades median: four persons [*P* < 0.0001]). The proportion of households with access to latrines and potable water sources was very high (> 99%) in both study areas (Supplemental Table 2).

### Trachoma clinical signs.

In Agdez, prevalence of TF among 1,246 one-to-nine-year-olds was 0.2% (95% CI: 0–0.5%), and no children had evidence of TI (Supplemental Table 2). Among 1,542 persons aged ≥ 15 years, TT prevalence was 0.6% (95% CI:0–1.6%), TS prevalence was 8.0% (95% CI: 3.3–15.2), and CO prevalence was 4.8% (95% CI: 1.1–10.0%). Two of the 16 individuals with TT also had TS (0.02%, 95% CI: 0–0.1%). In Boumalene Dades, prevalence of TF among 1,121 one-to-nine-year-olds was 0.1% (95% CI: 0–0.2%), and no children had evidence of TI. Among 1,121 persons aged ≥ 15 years, TT prevalence was 0.2% (95% CI:0–0.4%), TS prevalence was 3.2% (95% CI: 0.5–6.8%), and CO prevalence was 1.1% (95% CI: 0–3.3). None of the individuals with TT also had TS. A follow-up visit in June 2021 to the persons identified as having TT in this survey (*N* = 20 total) revealed that 15 did not have current signs of TT and may have been misdiagnosed, four were known to the public health system and refused surgery, and one was referred for and had corrective surgery. Supplemental Figure 1 shows overlap between each of the indicators in each district. Supplemental Table 3 shows the proportion of people with each sign that were seropositive in each district.

### Seroprevalence of antibodies against Pgp3 and CT94.

Among 2,917 participants from Agdez, age-adjusted seroprevalence was 36.2% (95% CI: 21.2–52.9%) by CT694 MBA, 29.7% (95% CI: 16.2–47.6%) by Pgp3 MBA, and 39.2% (95% CI: 23.4–55.1%) by Pgp3 LFA. Among 1,246 one-to-nine-year-olds, seroprevalence was 9.4% (95% CI: 5.9–15.6) by CT694 MBA, 11.4% (95% CI: 7.7–17.7%) by Pgp3 MBA, and 10.6% (95% CI: 6.9–16.9) by Pgp3 LFA. In Boumalene Dades, age-adjusted seroprevalence among 2,480 participants was 14.6% (95% CI: 5.0–42.3) by CT694 MBA, 14.0% (95% CI: 4.5–39.2%) by Pgp3 MBA, and 17.5% (95% CI: 6.4–41.8%) by Pgp3 LFA. Among 1,352 one-to-nine-year-olds, seroprevalence was 1.7% (95% CI:0.6–5.8) by CT694 MBA, 1.7% (95% CI: 0.6–5.7) by Pgp3 MBA, and 1.9% (95% CI:0.7–6.0) by Pgp3 LFA (Table 2). There was high agreement between the test results in this cohort using the Pgp3 and CT694 antigens (Kappa = 0.8, 95% CI: 0.8–0.9, *P* < 0.0001).

**Table 2:** Clinical and seroprevalence data by age group from Agdez and Boumalene Dades districts, Morocco

	Agdez									Boumalene Dades								
	1–9			> 15			Overall			1–9			> 15			Overall		
	*N* = 1,246			*N* = 1,542			*N* = 2,917			*N* = 1,352			*N* = 1,121			*N* = 2,480		
	n	%	(95% CI)	n	%	(95% CI)	n	%	(95% CI)	n	%	(95% CI)	n	%	(95% CI)	n	%	(95% CI)
Trachoma signs*																		
TF	3	0.2	(0‒0.5)	–	–	–	–	–	–	1	0.1	(0‒0.2)	–	–	–	–	–	–
TI	0	–	–	–	–	–	–	–	–	0	–	–	–	–	–	–	–	–
TS	70	5.8	(1.4‒12.6)	169	8.0	(3.3‒15.2)	250	7.11	(2.7‒14.9)	114	7.7	(1.0‒17.2)	49	3.2	(0.5‒6.8)	163	3.8	(0.7‒8.1)
TT	0	–	–	16	0.6	(0‒1.6)	16	0.41	(0‒1.1)	0	–	–	4	0.2	(0‒0.4)	4	0.1	(0‒0.3)
CO	50	4.3	(0.3‒9.3)	93	4.8	(1.1‒10.0)	155	4.55	(1.0‒9.7)	27	2.4	(0‒7.1)	15	1.1	(0‒3.3)	42	1.3	(0‒3.9)
Seroprevalence*†																		
CT694 MBA	138	9.4	(5.9‒15.6)	692	24.7	(13.6‒38.5)	849	36.2	(21.2‒52.9)	25	1.7	(0.6‒5.8)	136	12.0	(4.3‒33.1)	162	14.6	(5.0‒42.3)
Pgp3 MBA	171	11.4	(7.7‒17.7)	719	19.0	(10.4‒35.0)	712	29.7	(16.2‒47.6)	25	1.7	(0.6‒5.7)	133	11.6	(4.0‒33.1)	159	14.0	(4.5‒39.2)
Pgp3 LFA	156	10.6	(6.9‒16.9)	546	26.4	(15.2‒40.1)	896	39.2	(23.4‒55.1)	28	1.9	(0.7‒6.0)	170	15.1	(5.8‒35.7)	199	17.5	(6.4‒41.8)

CO = corneal opacity; LFA = lateral flow assay; MBA = multiplex bead assay; TF = trachomatous inflammation—follicular; TI = trachomatous inflammation—intense; TS = trachomatous scarring; TT = trachomatous trichiasis.

* Percentages and confidence intervals correspond to age-weighted estimates of cluster means for each district.

† Wilson’s score confidence intervals.

**Figure 2.**
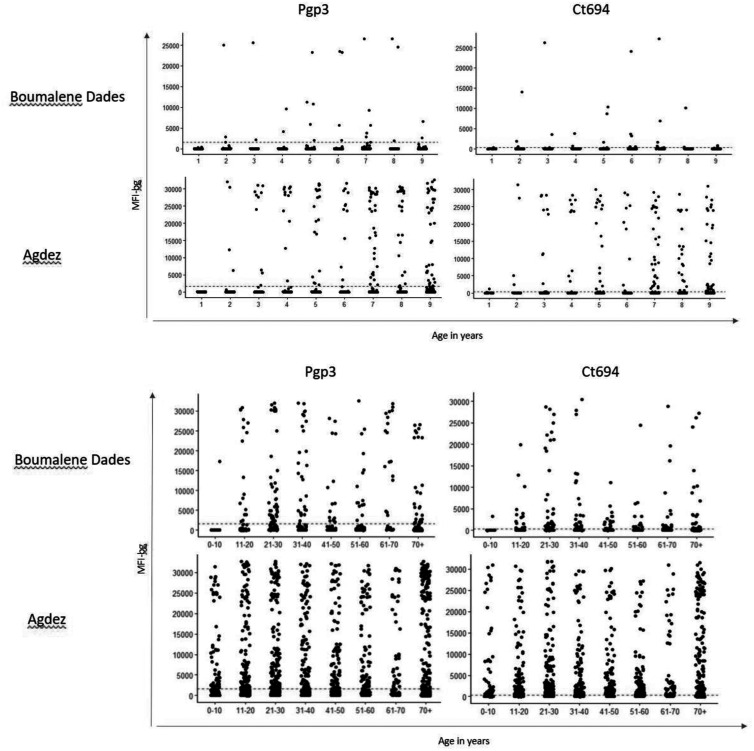
Intensity of antibody responses by year of age stratified by year of age in 1‒9-year-olds (top) or by decade of age in all ages (bottom). Data are shown for Boumalene Dades and Agdez for both *C. trachomatis* antigens (Pgp3 and CT694). Each point represents a single study participant. MFI-bg- median fluorescence intensity with background subtracted.

Intensity of antibodies in each specimen, represented by MFI, by year of age is shown in Figure 2. Among seropositive individuals of all ages, median MFI by CT694 MBA was significantly higher in Agdez (MFI = 2,837) compared with Boumalene Dades (MFI = 1,720) (*P* = 0.0018). Similarly, among seropositive 1–9-year-olds, median MFI by CT694 MBA was significantly higher in Agdez (MFI = 12,048) compared with Boumalene Dades (MFI = 2,536) (*P* = 0.00061). The median MFI by Pgp3 MBA was also higher among individuals of all ages in Agdez (MFI = 11,267) compared with Boumalene Dades (MFI = 9,746), although this difference was not statistically significant (*P* = 0.55). Among 1–9-year-olds, however, as was the case for the CT694 antibody, the median MFI by Pgp3 MBA in Agdez (MFI = 24,161) was significantly greater than in Boumalene Dades (MFI = 6,631) (*P* = 0.00037).

### Serocatalytic models.

**Figure 3.**
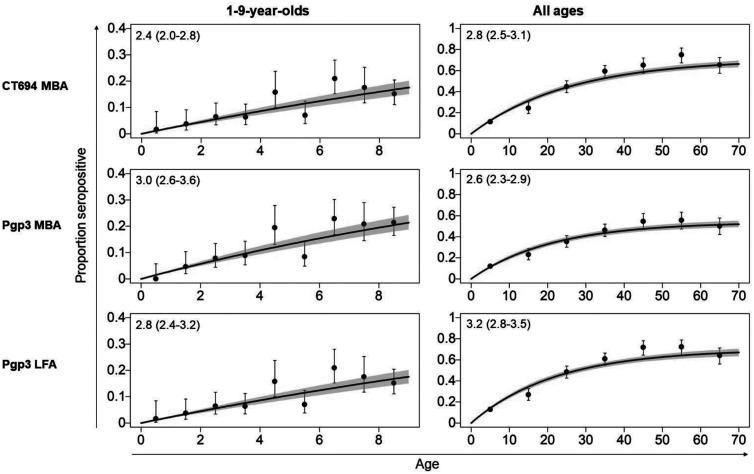
Seroprevalence curves for 1‒9-year-olds and participants of all ages—Agdez, Morocco. Black vertical lines represent 95% confidence intervals (Wilson’s score interval), and the purple-shaded regions represent the credible intervals. Solid purple lines represent the median parameter estimates from each model fit. Numbers in boxes show SCR with 95% credible intervals. A single force of infection for both 1‒9-year-olds and all-age data is shown. The y-axis scale ranges from 0 to 0.4 for 1‒9-year-olds and from 0 to 1 for all ages. LFA = lateral flow assay; MBA = multiplex bead assay.

**Figure 4.**
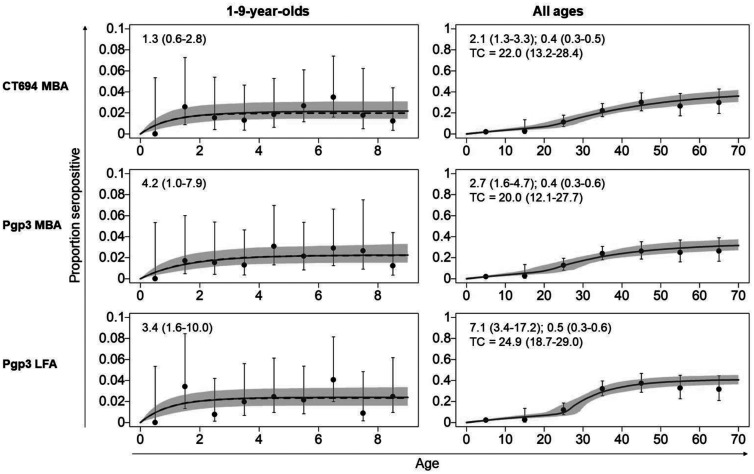
Seroprevalence curves for 1‒9-year-olds and participants of all ages—Boumalene Dades, Morocco. Black vertical lines represent 95% confidence intervals (Wilson’s score interval), and the purple shaded regions represent the credible intervals. Solid purple lines represent the median parameter estimates from each model fit. Numbers in boxes show SCR with 95% credible intervals. A single force of infection for both 1‒9-year-olds and all-age data is shown. The y-axis scale ranges from 0 to 0.1 for 1‒9-year-olds and from 0 to 1 for all ages. LFA = lateral flow assay; MBA = multiplex bead assay; SCR = seroconversion rates.

Parameter estimates for each of the sero-catalytic models are shown in Table 3 (1–9-year-olds, constant transmission) and Table 4 (all ages, constant transmission). Seroconversion rates in 1–9-year-olds ranged from 2.4 to 3.0 per 100 children per year in Agdez (Figure 3) and 0.4–0.5 per 100 children per year in Boumalene Dades (Figure 4). Estimated SRR in children were similar in Boumalene Dades (1.7–2.7 per 100 children per year) compared with Agdez (1.7–2.6 per 100 children per year) (Table 3). Markov chain Monte Carlo (MCMC) chain diagnostics are shown in Supplemental Table 4.

**Table 3:** Serocatalytic constant transmission model results, children aged 1–9

Study site	Test	λT	(95% CrI)	p (rho)	(95% CrI)
Boumalene Dades	CT694 MBA	0.4	(0.3‒0.6)	1.7	(1.3‒2.2)
	Pgp3 MBA	0.4	(0.3‒0.6)	2.7	(2.0‒3.2)
	Pgp3 LFA	0.5	(0.3‒0.7)	2.6	(1.9‒3.3)
Agdez	CT694 MBA	2.4	(2.0‒2.8)	1.7	(1.3‒2.1)
	Pgp3 MBA	3.0	(2.6‒3.6)	2.6	(2.0‒3.2)
	Pgp3 LFA	2.8	(2.4‒3.2)	2.6	(2.0‒3.2)

CrI = credible interval; LFA = lateral flow assay; MBA = multiplex bead assay; λT = rate of seroconversion due to exposure to trachoma; ρ = rate of sero-reversion. Model parameters λT and p were scaled *100 for ease of interpretation.

**Table 4:** Serocatalytic modeling results, all ages

Study site	Test	Model	λT	(95% CrI)	λc	(95% CrI)	γ	(95% CrI)	p (rho)	(95% CrI)	Time_c	(95% CrI)
Boumalene Dades	CT694 MBA	2	2.1	(1.3‒3.3)	0.4	(0.3‒0.5)	0.2	(0.1‒0.4)	1.7	(1.3‒2.1)	22.0	(13.2‒28.4)
	Pgp3 MBA	2	2.7	(1.6‒4.7)	0.4	(0.3‒0.6)	0.2	(0.1‒0.3)	2.6	(2.1‒3.2)	20.0	(12.1‒27.7)
	Pgp3 LFA	2	7.1	(3.4‒17.2)	0.5	(0.3‒0.6)	0.1	(0.02‒0.2)	2.7	(2.1‒3.2)	24.9	(18.7‒29.0)
Agdez	CT694 MBA	1	2.8	(2.5‒3.1)	–	–	–	–	1.1	(0.9‒1.5)	–	–
	Pgp3 MBA	1	2.6	(2.3‒2.9)	–	–	–	–	2.2	(1.8‒2.7)	–	–
	Pgp3 LFA	1	3.2	(2.8‒3.5)	–	–	–	–	1.4	(1.0‒1.9)	–	–

CrI = credible interval; MBA = multiplex bead assay; LFA = lateral flow assay; Time_c = time of change of transmission; λT = rate of seroconversion due to exposure to trachoma; λc = rate of seroconversion due to exposure to trachoma, following the identified fixed time point at which transmission intensity changed (time_c); ρ = rate of sero-reversion; γ = proportional decline in transmission at time_c or over time.

In Agdez, all-age seroprevalence data showed that Model 1 (constant transmission) was a better fit for the data because significant autocorrelation was observed in Model 2 (Full results for both models and MCMC diagnostics are shown in Supplemental Table 5) SCR in all ages ranged from 2.6–3.2 per 100 people per year in Agdez (Figure 3). In Boumalene Dades, Model 2 (fixed time point) was a better fit for the data based on lower deviance information criteria (DIC) estimates. The time point of change ranged from 20.0 to 24.9 years (Figure 4). The SCR estimates before the change point ranged from 2.1 to 7.1 while the SCR estimates after the change point ranged from 0.4 to 0.5. SRR was similar in both study sites (Agdez: 1.1‒2.2 per 100 people per year; Boumalene Dades: 1.7‒2.7 per 100 people per year).

## DISCUSSION

We present here a population-based post-validation survey for trachoma that includes evaluation of serology as an alternative indicator. In each of the surveyed districts, TF was maintained below the 5% threshold for elimination as a public health problem. Because countries will likely not have funding to surveil previously endemic districts for re-emergent ocular *C. trachomatis* using clinical signs, we have been evaluating serology as a potential tool for post-elimination surveillance of trachoma. In these surveys, we included antibody testing in 1–9-year-olds and observed distinctly different serological outcomes in two districts that had TF prevalence < 5% that was consistent between the three antibody tests used. The LFA may be a more sustainable approach to post-validation antibody testing, so this agreement between tests further supports the potential use of this assay. In Boumalene Dades, the low TF prevalence was reflected in low antibody prevalence (2%) and seroconversion rates (approximately < 1 seroconversion event/person/year). Agdez, which also had low TF prevalence, had antibody prevalence of 12% and a SCR of around 2–3, both higher than expected based on models derived data from multiple sites for which the predicted Ab prevalence was 6.5% and SCR of 1.5 corresponding to TF of < 5%.^25^ It is important to note that these are preliminary threshold estimates and that these data from Morocco and other more recent studies^16^^,^^17^^,^^29^ will be used to enhance and improve these models. However, it is worth trying to understand why such high levels of antibody positivity were seen in Agdez.

In contrast to low TF prevalence in each district, TT was higher than the 0.2% TT elimination targets set by WHO. The 2018 Fourth Global Scientific Meeting for Trachoma recommended that in cases where TT was seen without evidence of ongoing transmission additional etiologies for TT should be investigated. Among these is coincidence of TS, which would give strong support for a trachoma-based etiology of trichiasis. In Agdez, 2 of 16 individuals with TT also had TS, whereas in Boumalene Dades none of the four cases of TT also had TS. While this suggests no incident TT cases were detected in Boumalene Dades, TT cases were higher than expected in Agdez, even after factoring in coincidence of TS. Ensuring a system to deal with incident TT cases is an important part of the WHO validation dossier that may need periodic review by countries in a post-validation setting.

There are historical differences in trachoma prevalence in these districts that are reflected in several of the indicators assessed in this study. Zagora, where Agdez district is located, had much higher TF prevalence than Tinghir, where Boumalene Dades district is located: in 1997, Zagora had TF prevalence of 60% compared with 5% for Ouarzazate province (from which Tinghir split in 2009).^4^ Agdez was also slower to reach elimination that Boumalene Dades (2005 versus pre-2003, respectively). All-age antibody positivity was much higher in Agdez than in Boumalene Dades, suggesting relatively long-lived antibody responses due to repeated childhood infection with ocular *C. trachomatis*. Agdez also had higher TT prevalence in adults than BD, and in fact TT cases with TS were above the elimination threshold. Since TT lags the peak of infection and TF by decades, it would be expected that a district which previously had higher TF in children would later have higher TT in adults.

This study has several limitations. Infection was not measured, and this would have been a “tiebreaker” in Agdez to clarify if antibody positivity was associated with high rates of ocular infection or if it was nonspecific, This nonspecificity could be exposure to environmental chlamydial exposure, infection via delivery, or *C. pneumoniae* infection. There were < 5 DBS collected from 10 to 14-year-olds in Boumalene Dades, making all-age SCRs and changes in transmission (based on the “TC” time of change measure in serology models) difficult to estimate in this district. Age weights were developed at provincial rather than district scale, so age-adjusted estimates are not sensitive to demographic differences by district. While graders were trained ophthalmologists who have experience with the trachoma elimination efforts in Morocco, there was no formal Tropical Data-based standardized training. Finally, we only surveyed two districts and not all previously endemic areas; guidance for post-validation surveys, such as if all previously endemic districts need to be surveyed, has yet not been established.

Here we describe two post-validation settings with TF < 5% but with very different anti-*C. trachomatis* antibody profiles, but there is no clear explanation for this discrepancy between districts. It is interesting but not definitive that the district with higher Ab prevalence, Agdez, more recently eliminated trachoma and had a higher TF level 30 years ago. But with elimination occurring in 2005 in Zagora and < 1% TF in the current survey, one would expect that antibody positivity in young children would be negligible here, not approximately 11%. In the Solomon Islands, antibody positivity of approximately 11% in young children with little ocular infection was likely explained by a high prevalence of urogenital *C. trachomatis* infection in women of reproductive age (approximately 20%^30^). Recorded rates of sexually transmitted infections, including *Chlamydia*, are generally very low in Morocco based on quarterly syndromic surveillance and does not differ between Agdez and Boumalene Dades, although data on sexually transmitted infections may be underreported due to self-medication and/or lack of healthcare seeking in rural areas. Data on urogenital *Chlamydia* infection are not granular enough to directly compare with the trachoma serological data in this study, so the contribution of transient exposure to urogenital *Chlamydia* strains to the higher-than expected serological markers in Agdez.

The specificity of the Pgp3 immunoassay is approximately 98% ± 2%,^12^ so we do not expect to see a complete lack of antibody signal in a population. In post-elimination settings such as Nepal and Ghana, the antibody prevalence in children was < 5%.^17^^,^^18^ By contrast, districts with TF of just 7.5% have antibody positivity > 25%.^16^^,^^31^ Based on data from > 15 countries from which *C. trachomatis-*specific antibody data has been collected, anti-Pgp3 positivity of 10–20% in children is challenging to interpret, typically only seen when TF may not represent trachoma^32^ or in areas with declining TF as they approach elimination.^29^ An SCR approaching three per 100 children per year is similarly challenging to interpret. The preliminary estimates of SCR corresponding to < 5% TF is 1.5 seroconversion events per 100 children per year (95% CrI: 0–4.9).^25^ The SCR seen in Agdez falls within this range, so perhaps simply represents the high end of a distribution of this transmission marker. Incorporating data from this and other more recent studies^16^^,^^17^^,^^29^^,^^31^ into a meta-analysis will improve these estimates and begin creation of a decision tree determining what actions will be precipitated by different levels of seropositivity and/or seroconversion rates, should the preponderance of data suggest antibodies could be a useful tool for trachoma surveillance.

## Supplemental Material


Supplemental materials
