# Supplementary material for: Post-Validation Survey in Two Districts of Morocco after the Elimination of Trachoma as a Public Health Problem
Source: Am J Trop Med Hyg. 2022 Mar 28;106(5):1370–8. doi: 10.4269/ajtmh.21-1140 (PMC9128706; doi:10.4269/ajtmh.21-1140)
Supplement: Supplementary file 1 [file tpmd211140.SD1.pdf]

Supplemental Figure S1. Overlap in trachoma indicators in Agdez and Boumalene Dades districts, Morocco.

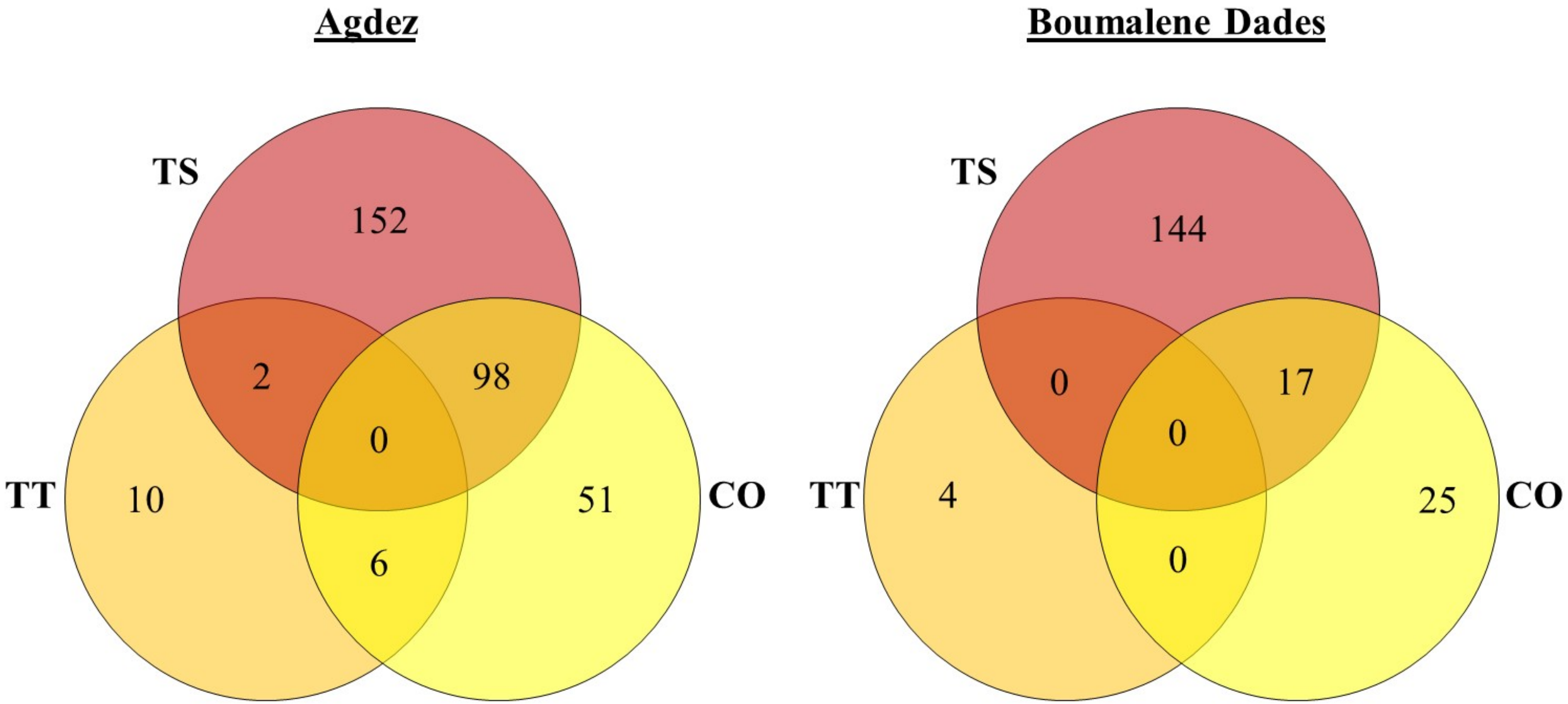

Supplemental Table S1. Summary of transmission models.

**Model Name**	**Transmission Assumption**	**λT**	**λc**	**ρ**	**tc**	**γ**
Scenario 1	Constant	Yes	No	Yes	No	No
Scenario 2	Fixed change point	Yes	Yes	Yes	Yes	Yes

λT Rate of seroconversion due to exposure to trachoma.
λc Rate of seroconversion due to exposure to trachoma, following the identified fixed time point at which transmission intensity changed (tc).
ρ Rate of sero-reversion.
tc Fixed time point at which transmission intensity changed.
γ Proportional decline in transmission at tc or over time. Ratio between average transmission rates at two time intervals.

Supplemental Table S2. Household-level characteristics in Agdez and Boumalene Dades districts, Morocco.

	Overall		Agdez (Zagora)		Boumalene Dades (Tinghir)		
	N = 1,213		n = 515 (42.5%)		n = 698 (57.5%)		
	**N**	**(%)**	**n**	**(%)**	**n**	**(%)**	**P***
**Access to a latrine**							
Yes	1,206	(99.7)	515	(100)	691	(99.4)	
No	4	(0.3)	0	(0)	4	(0.6)	
Missing	3		0		3		0.14
**Access to potable water**							
Yes	1,208	(99.8)	515	(100)	693	(99.7)	
No	2	(0.2)	0	(0)	2	(0.3)	
Missing	3		0		3		0.51

*Fisher's exact test p-value.

Supplemental Table S3. Serologic test results and trachoma indicators in Agdez and Boumalene Dades districts, Morocco.

	Agdez								Boumalene Dades							
	1-9				>15				1-9				>15			
		Pgp3	CT694	LFA		Pgp3	CT694	LFA		Pgp3	CT694	LFA		Pgp3	CT694	LFA
	**N***	**n (%)**	**n (%)**	**n (%)**	**N***	**n (%)**	**n (%)**	**n (%)**	**N***	**n (%)**	**n (%)**	**n (%)**	**N***	**n (%)**	**n (%)**	**n (%)**
TF	3	2 (66.67)	3 (100)	3 (100)	--	- -	- -	--	1	0 (0)	0 (0)	0 (0)	--	- -	- -	- -
TI	--	--	--	- -	--	- -	- -	--	--	- -	- -	- -	--	- -	- -	- -
TS	28	2 (7.14)	2 (7.14)	2 (7.14)	96	52 (54.17)	55 (57.29)	62 (64.58)	114	5 (4.39)	4 (3.51)	3 (2.63)	49	17 (34.69)	19 (38.78)	23 (46.94)
TT	--	--	--	- -	14	6 (42.86)	6 (42.86)	7 (50)	--	- -	- -	- -	2	2 (100)	2 (100)	2 (100)
CO	24	2 (8.33)	2 (8.33)	2 (8.33)	42	20 (47.62)	24 (57.14)	29 (69.04)	27	4 (14.81)	4 (14.81)	5 (18.52)	15	2 (13.33)	1 (6.67)	2 (13.33)

*Serologic test available among persons with each trachoma indicator. MBA: multiplex bead assay.; LFA: lateral flow assay; TF: trachomatous inflammation—follicular; TI: trachomatous inflammation—intense; TS: trachomatous scarring; TT: trachomatous trichiasis; CO: corneal opacity.

Supplemental Table S4. Serocatalytic constant transmission model and model diagnostics, children 1-9.

Study Site	Test	λT	(95% CrI)	GR	ESS	p (rho)	(95% CrI)	GR	ESS	DIC
Boumalene Dades	CT694 MBA	0.4	(0.3−0.6)	1.03	486.5	1.7	(1.3−2.2)	1.01	452.8	248.5
	Pgp3 MBA	0.4	(0.3−0.6)	1.01	726.3	2.7	(2.0−3.2)	1.00	623.7	246.4
	Pgp3 LFA	0.5	(0.3−0.7)	1.00	661.2	2.6	(1.9−3.3)	1.00	582.8	271.1
Agdez	CT694 MBA	2.4	(2.0−2.8)	1.01	945.5	1.7	(1.3−2.1)	1.01	1083.2	827.2
	Pgp3 MBA	3.0	(2.6−3.6)	1.00	1781.1	2.6	(2.0−3.2)	1.01	1656.1	939.4
	Pgp3 LFA	2.8	(2.4−3.2)	1.01	1627.2	2.6	(2.0−3.2)	1.00	1106.0	891.6

Model parameters λT and p were scaled *100 for ease of interpretation.
CrI: Credible Intervals; MBA: multiplex bead assay; LFA: lateral flow assay; GR: Gelman-Rubin statistic; ESS: Effective sample size; DIC: Deviance information criterion; λT: rate of seroconversion due to exposure to trachoma; ρ: rate of sero-reversion.

Supplemental Table S5. Serocatalytic model diagnostics, all ages.

Study Site	Test	Model	λT	(95% CrI)	GR	ESS	λc	(95% CrI)	GR	ESS	γ	(95% CrI)	GR	ESS	p (rho)	(95% CrI)	GR	ESS	time_c	(95% CrI)	GR	ESS	DIC
Boumalene Dades	CT694 MBA	1	0.7	(0.6−0.9)	1.01	494.8									1.5	(1.1−1.9)	1.00	504.4					886.3
	CT694 MBA	2*	2.1	(1.3−3.3)	1.00	845.9	0.4	(0.3−0.5)	1.00	1426.3	0.2	(0.1−0.4)	1.00	813.3	1.7	(1.3−2.1)	1.00	1362.3	22.0	(13.2−28.4)	1.00	1025.0	861.2
	Pgp3 MBA	1	0.8	(0.7−0.9)	1.03	519.5									2.1	(1.6−2.6)	1.03	461.1					887.9
	Pgp3 MBA	2*	2.7	(1.6−4.7)	1.02	643.8	0.4	(0.3−0.6)	1.03	1248.5	0.2	(0.1−0.3)	1.06	565.1	2.6	(2.1−3.2)	1.00	1181.0	20.0	(12.1−27.7)	1.01	900.7	864.4
	Pgp3 LFA	1	1.0	(0.8−1.2)	1.01	643.3									1.8	(1.4−2.4)	1.01	508.5					995.1
	Pgp3 LFA	2*	7.1	(3.4−17.2)	1.03	488.5	0.5	(0.3−0.6)	1.00	2716.5	0.1	(0.02−0.2)	1.01	799.8	2.7	(2.1−3.2)	1.00	1415.4	24.9	(18.7−29.0)	1.01	1222.7	945.2
Agdez	CT694 MBA	1*	2.8	(2.5−3.1)	1.01	440.3									1.1	(0.9−1.5)	1.00	440.3					2548.6
	CT694 MBA	2	7.8	(4.8−15.1)	1.12	103.1	2.3	(2.0−2.7)	1.01	1770.9	0.3	(0.2−0.5)	1.04	235.0	1.6	(1.3−1.9)	1.03	410.8	21.7	(16.3−32.4)	1.03	318.5	2530.0
	Pgp3 MBA	1*	2.6	(2.3−2.9)	1.00	636.0									2.2	(1.8−2.7)	1.02	638.8					2610.4
	Pgp3 MBA	2	4.9	(2.7−6.2)	1.36	21.1	2.4	(0.8−2.7)	1.00	539.1	0.5	(0.3−0.9)	1.31	25.7	2.5	(2.0−3.0)	1.03	675.7	21.8	(1.0−31.2)	1.07	61.8	2605.1
	Pgp3 LFA	1*	3.2	(2.8−3.5)	1.01	602.6									1.4	(1.0−1.9)	1.01	644.9					2616.0
	Pgp3 LFA	2	11.9	(7.1−24.1)	1.54	96.4	2.7	(2.3−3.1)	1.03	1618.0	0.2	(0.1−0.4)	1.24	212.8	2.0	(1.6−2.5)	1.00	425.3	21.6	(17.7−31.5)	1.20	218.3	2597.3

*Selected model, based on lower DIC (for Boumalene Dades) or lack of autocorrelation (for Agdez)

Model parameters λT, λc, and p were scaled *100 for ease of interpretation.

CrI: Credible Intervals; MBA: multiplex bead assay; LFA: lateral flow assay; GR: Gelman-Rubin statistic; ESS: Effective sample size; DIC: Deviance information criterion; λT: rate of seroconversion due to exposure to trachoma; λc: rate of seroconversion due to exposure to trachoma, following the identified fixed time point at which transmission intensity changed (time_c); ρ: rate of sero-reversion; γ: proportional decline in transmission at time_c or over time.
